# A systematic review on machine learning and deep learning techniques in the effective diagnosis of Alzheimer’s disease

**DOI:** 10.1186/s40708-023-00195-7

**Published:** 2023-07-14

**Authors:** Akhilesh Deep Arya, Sourabh Singh Verma, Prasun Chakarabarti, Tulika Chakrabarti, Ahmed A. Elngar, Ali-Mohammad Kamali, Mohammad Nami

**Affiliations:** 1 SCIT, Manipal University Jaipur, Jaipur, 303007 India; 2 ITM (SLS), Baroda University, Vadodara, Gujarat 391510 India; 3 Sir Padampat Singhania University, Udaipur, Rajasthan 313601 India; 4 Faculty of Computers and Artificial Intelligence, Beni-Suef University, Beni-Suef, 62511 Egypt; 5 Department of Neuroscience, School of Advanced Medical Sciences and Technologies, Shiraz University of Medical Sciences, Shiraz, Iran; 6 Cognitive Neuropsychology Unit, Department of Social Sciences, Canadian University Dubai, Dubai, UAE

**Keywords:** Alzheimer’s disease, Dementia, MCI, Neurodegenerative, Machine learning, Deep learning

## Abstract

Alzheimer’s disease (AD) is a brain-related disease in which the condition of the patient gets worse with time. AD is not a curable disease by any medication. It is impossible to halt the death of brain cells, but with the help of medication, the effects of AD can be delayed. As not all MCI patients will suffer from AD, it is required to accurately diagnose whether a mild cognitive impaired (MCI) patient will convert to AD (namely MCI converter MCI-C) or not (namely MCI non-converter MCI-NC), during early diagnosis. There are two modalities, positron emission tomography (PET) and magnetic resonance image (MRI), used by a physician for the diagnosis of Alzheimer’s disease. Machine learning and deep learning perform exceptionally well in the field of computer vision where there is a requirement to extract information from high-dimensional data. Researchers use deep learning models in the field of medicine for diagnosis, prognosis, and even to predict the future health of the patient under medication. This study is a systematic review of publications using machine learning and deep learning methods for early classification of normal cognitive (NC) and Alzheimer’s disease (AD).This study is an effort to provide the details of the two most commonly used modalities PET and MRI for the identification of AD, and to evaluate the performance of both modalities while working with different classifiers.

## Introduction

Alzheimer’s disease (AD) is a progressive neurodegenerative disease in elderly people which causes impairment of cognitive and memory function. A person suffering from this disease finds it difficult to perform routine tasks. Not able to remember maps and sometimes the name of a person. For the past few years, many research groups are trying and making efforts for early detection of AD using different machine learning models.

There are various risk factors for AD some of them are age, genetics, education, and coexisting health problems [1]. Most cases of this disease are observed in people aging 65 and above. While the percentage of AD patients between the ages of 65 and 74 is 5%, the risk increases by 50% above the age of 85. It is observed that people with higher education are at less risk. Higher education leads to the formation of more synaptic connections in the brain. This creates a synaptic reserve in the brain, enabling patients to compensate for the loss of neurons as the disease progresses [1, 2]. One of the major causes of AD is coexisting health problems. It is observed that people suffering from cardiovascular disease, high blood pressure, or type-2 diabetes increase the risk of Alzheimer’s disease [2].

There is no medication available to cure and prevent Alzheimer's disease. The only way to lower the risk of Alzheimer’s disease is by decreasing the risk of heart disease. It is observed that people at a high risk of heart disease are also at a high risk of Alzheimer’s disease. Major factors include excess weight, high blood pressure, diabetes, and high cholesterol. As no proper treatment is available to cure and prevent Alzheimer's disease the only way is to maintain the mental function of the patient and with some medication delays the symptoms of Alzheimer’s disease. Early prediction of Alzheimer’s disease is really helpful to a doctor to maintain the mental health of the patient and delays the effect which helps the patient to live a better life [2].

**Fig. 1:**
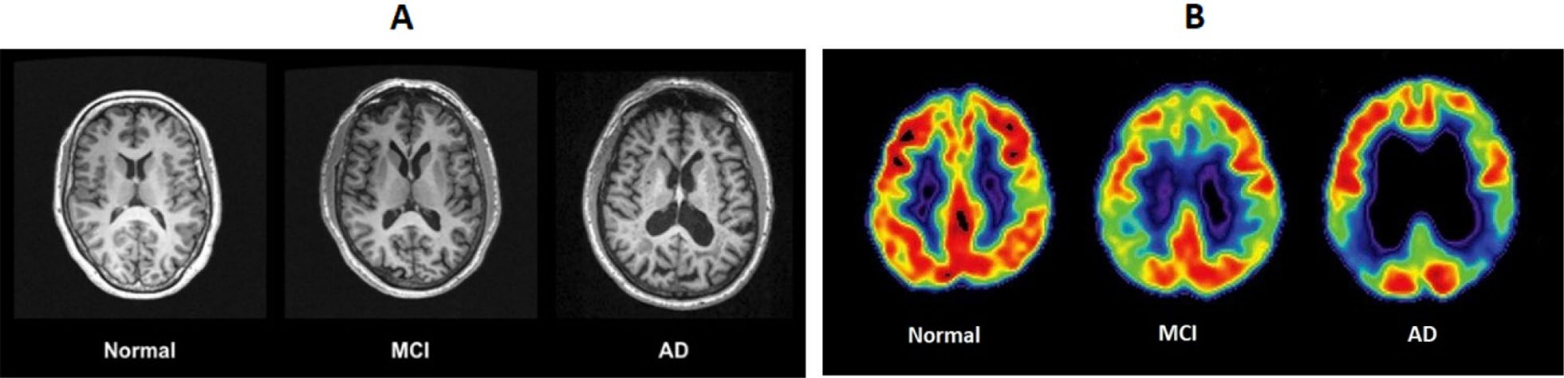
**A** MRI scans images of different stages of AD. **B** PET scan images of different stages of AD

There are five stages associated with AD as shown in Table 1. Figure 1 represents the MRI and PET scans of different stages of AD. In the effective prediction of Mild Cognitive Impairment 25 (MCI) two main factors are to be considered, the first is the qualitative change (i.e., progression of AD) and the second one is a quantitative change which includes the cognitive scores. Qualitative changes can be measured using classification between the MRI images of MCI-C (Mild Cognitive Impairment convertor) and MCI– NC (Mild Cognitive Impairment non-convertor). To calculate quantitative changes, clinical scores, e.g., Mini-Mental State Examination (MMSE) and Alzheimer’s disease Assessment’s scale- Cognitive Subscale (ADAS- cog.) are measured at different points of time. Disease progression is measured based on the clinical scores at the previous point in time [3].

**Table 1:** Different stages of MCI/AD

There are five stages associated with Alzheimer's disease:	
1	Preclinical Alzheimer's disease
2	Mild cognitive impairment (MCI)
3	Mild dementia
4	Moderate dementia
5	Severe dementia due to Alzheimer's disease

High diagnostic accuracy can be achieved by the estimation of functional connectivity of the brain using functional Magnetic Resonance Image (fMRI). As fMRIs are high dimensional in nature, this is always a challenge to estimate functional connectivity, but this can be done by using discriminative group sparse representation [4].

Studies have shown that computer vision and deep learning models have shown quite good results in diagnosing viral [5] or non-viral diseases using medical imaging. In AD, an image processing framework can be used to track changes in white matter at different points of time using diffusion tensor imaging (DTI). This helps to find new insights into the progression of AD. A pipeline is created 40 by 2 scans of a subject at two different times and using test–reset methods and bootstrap method high precision in prognosis is achieved [6]

Multiple methods have been attempted to predict prevalence at the outset of AD. The graph-based multiple instances learning method used pre-processed images where patches were used as features for classification. This mi-Graph-based method provides results that are consistently better than the traditional approach to image processing [7].

Taeho Jo. et al. 2019 [8] presented a comparative study of traditional machine learning methods and deep learning methods in the early detection of AD and progression of MCI to AD. They considered 16 studies out of which 4 were using deep learning approaches and traditional machine learning together and 12 studies were using only deep learning approaches. With the use of a deep learning 50 approach with the traditional machine learning approach, the efficiency of 96.0% for feature selection and 84.2% for MCI to AD conversion is achieved. Using convolutional neural networks (CNN) in the deep learning approach the accuracy of 96.0% is achieved in feature selection and an accuracy of 84.2% is achieved in MCI to AD conversion prediction. In addition to that, it is observed that performance in the classification can be improved by using multimodal neuroimaging with fluid biomarkers.

Garam Lee et al. [9] use multimodal recurrent neural networks (RNN) to identify the progression of MCI to AD. Cross-sectional neuroimaging, longitudinal CSF analysis, and cognitive performance were used in the proposed framework to improve prediction accuracy. The study found that using a single modality, the accuracy achieved was 75%, whereas using multiple modalities increased the accuracy to 81%.

Zhe Xiao et al. 2017 proposed a framework that extracts multiple features from MRI to train a multi-featured model. This model can effectively identify the subjects suffering from AD. It was noticed that the multi-featured model performs better in comparison to the single feature model [10].

Andres Ortiz et al. use a deep learning approach to classify AD, MCI, and non-converting (NC) subjects. They used automated anatomical labeling (AAL) [11] software package to split-brain the area into 3D patches; these patches were used to train deep neural networks. For the prediction part, four voting algorithms were used and the results were compared. This classification model provided an accuracy value of 0.90 in NC/ AD classification [12].

There are certain limitations of the current machine learning techniques in the prediction of AD. One of them is missing data exclusion. Lei Huang et al. [13] proposed a soft split technique to find the missing scores of the subject and use them at all the previous times to predict the scores the next time. The model performs well and the results are better than the previous studies.

Subramoniam et al. [14] proposed a model where they slice the MRI images and use them as input to the residual convolutional neural networks (ResNet-101) for feature extraction and classification. This model classifies and labels images into four classes moderately demented, mild demented, very mild demented, and non-demented. Using 3 layers of CNN followed with the 3 layers of Vanilla-dense neural network (DNN) this model achieves an accuracy of 95.32%. In DNN first 2 dense layers are used with rectified linear unit (ReLU) activation and 1 unit of dense layer with softmax activation.

Edward challis et al. [15] compared SVM classifiers and Gaussian process logistic regression (GP-LR) and found that the GP-LR method performs well in comparison to SVM. SVM provides a binary classifier, whereas GP-LR provides the probability of class membership. During the study a total of 77 subjects were taken out of which 27 were AD patients, 50 were suffering from MIC, and 39 were controlled subjects. Accuracy of 75% is achieved in the control of MCI conversion whereas 97% in the case of MCI to AD is achieved.

Alzheimer's disease (AD) is a progressive neurodegenerative disease that affects the cognitive and memory function of elderly people and its early detection is crucial for delaying its effects on the patient's mental health. Several risk factors, including age, genetics, education, and coexisting health problems, increase the risk of AD. There is no medication available to cure AD, but maintaining the mental function of the patient and delaying the symptoms is the only way to lower the risk. Computer vision and deep learning models have shown quite good results in diagnosing AD using medical imaging, multiple methods explained above have been attempted to predict prevalence at the outset of AD. The most favored method among researchers for predicting Alzheimer's disease onset using medical imaging is using fMRI/PET scan data with an SVM classifier and CNN [16]. This study presents a statistical analysis of the different machine learning and deep learning models proposed by researchers, along with the various modalities employed.

The paper is structured as follows: Sect. 2 outlines the study's material and methods, including the selection process of relevant literature using the Preferred Reporting Item for Systematic Review and Meta-Analysis (PRISMA) flowchart [16], the different datasets used, data pre-processing techniques, various machine learning and deep learning techniques used, and performance evaluation metrics considered for the evaluation. Section 3 presents the experimental details and comparison results. Section 4 discusses the limitations of the study, while Sect. 5 concludes with a future outlook.

## Material and methods used

In this section, selection procedure of the literature for review, various methods used in different studies, and the dataset used by the researchers are discussed.

### Literature selection

**Fig. 2:**
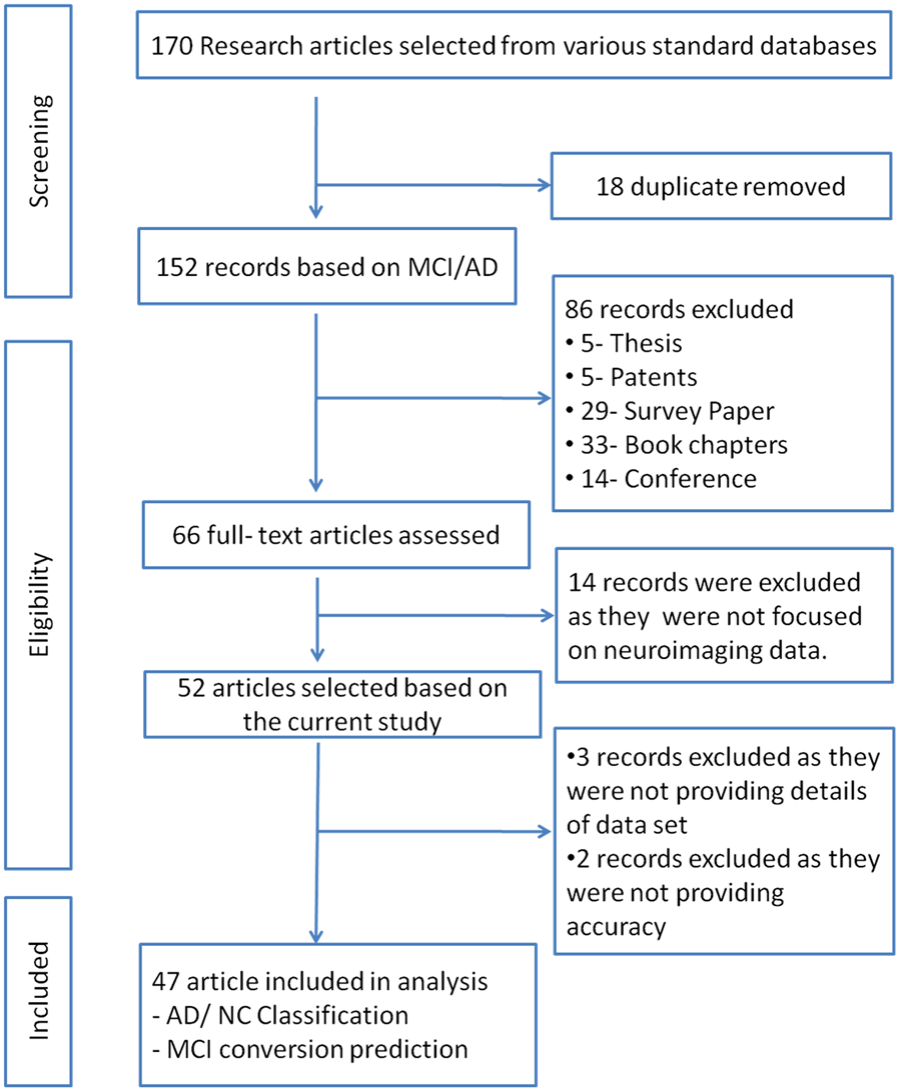
Preferred Reporting Item for Systematic Review and Meta-Analysis (PRISMA) flowchart. The figure explains the selection procedure of literature used for the study

A systematic review process is performed using the previous publications on the diagnosis and prognosis of Alzheimer’s disease and MCI conversion prediction using machine learning and deep learning approaches. During the survey process, the research articles using MRI/PET scans and clinical dataset as modalities are considered in the review. A total of 47 papers were selected and included in the review; out of which 31 papers are used for the comparative analysis and 16 were used for the literature background. The selection procedure is explained using the preferred reporting item for systematic review and meta-analysis (PRISMA) flowchart in Fig. 2.

### Dataset

There are 3 major sources of the dataset considered in this study:

- ADNI: Alzheimer’s disease neuroimaging initiative started in the year 2004 to provide researchers with neuroimages for the effective diagnosis and prognosis of Alzheimer’s disease under the leadership of Dr. Michael W. Weiner. 50% of the research articles included in the study are using 1.5 T, 3 T MRI, fMRI images and FDG-PET images as a dataset [9].
- OASIS: Open access series of imaging studies is started with the aim to provide a free neuroimaging dataset to the research community. It provides 3 kinds of data sets:

a. OASIS-1: contains the study of 416 subjects with the 434 cross-sectional MRI data set of demented and non-demented older adults.

b. OASIS-2: contains the study of 150 subjects with the 373 longitudinal MRI datasets of demented and non-demented older adults.

c. OASIS-3: contains the study of 1098 subjects with 2168 MRI and 1608 PET images.

35% of the research articles included in the study are using the OASIS dataset.

- Kaggle: offers data set for various research-oriented studies 15% of the research articles use the dataset from Kaggle [13].

Table 3 contains the details such as source, modality, and other numbers of demented and non-demented subjects in the dataset used by different research articles.

### Pre-processing of data

To prepare a clean data set various data pre-processing strategies are used in the studies.

Research publications used in this review paper are using two types of data set: one is the clinical data set from OASIS and the second one is image dataset from ADNI and kaggle platform as explained in Sect. 2.2.

There are various data pre-processing methods that can be applied to clinical data sets of Alzheimer's disease to improve the quality and accuracy of the analysis results. Some of the most commonly used pre-processing methods used by the researchers are:

- **Data cleaning:** This involves removing or correcting any incomplete, inconsistent, or erroneous data from the dataset. This is typically done by identifying missing values, outliers, and inconsistencies in the data, and then removing or replacing them [8].
- **Feature selection**: This involves selecting the most relevant features from the dataset that are likely to have a significant impact on the analysis results. This can be done using various statistical techniques such as correlation analysis, principal component analysis, and mutual information [14].
- **Data transformation:** This involves transforming the data into a more suitable format for analysis. This can be done using techniques such as normalization, standardization, and logarithmic transformation [6].
- **Imputation:** This involves filling in missing data with estimated values. This can be done using various imputation techniques such as mean imputation, regression imputation, and multiple imputations.

Image dataset for Alzheimer's consist of MRI ad PET images. Pre-processing methods used in the research articles that were used in this review article are:

- **Image normalization:** This is the process of adjusting the intensity values of images to a common scale. This can be done using techniques such as histogram equalization, contrast stretching, and normalization to improve the visual quality of the images [12].
- **Image cropping and resizing:** This involves resizing images to a uniform size and cropping out any unnecessary parts of the image that do not contain relevant information. This can help reduce the computational complexity of the analysis and improve the efficiency of the algorithms.
- **Image augmentation:** This involves generating additional images from the original dataset by applying various transformations such as flipping, rotating, and scaling. This can help increase the size of the dataset and improve the robustness of the analysis [14].
- **Feature extraction:** This involves extracting relevant features from the images that can be used in the analysis. This can be done using techniques, such as edge detection, texture analysis, and shape analysis [10].
- **Data augmentation:** This involves increasing the size of the dataset by adding variations of the original images, such as changing brightness or contrast, or adding noise [8].

### Conventional machine learning models used

A) Logistic regression

Logistic regression is the type of supervised machine learning algorithm. This algorithm is useful in cases where we have to predict the outcome in binary (true or false). The equation of the logistic regression is:

1 $$p = {1 \mathord{\left/ {\vphantom {1 {\left( {1 + e^{ \wedge } ( - (b_{0} + b_{1} x + b_{2} x^{ \wedge } 2))} \right)}}} \right. \kern-\nulldelimiterspace} {\left( {1 + e^{ \wedge } ( - (b_{0} + b_{1} x + b_{2} x^{ \wedge } 2))} \right)}}$$

where *'e'* is Euler's number which is 2.71828, b0, b1, and, b2 are the coefficient of the model, bo is the intercept, while '*b1'* and '*b2'* are the slope of the liner and quadratic terms of the predictor variable *'x'*. These coefficients are estimated from the training data using method called as maximum likelihood estimation.

- B) Support vector machine

SVM is a fast classification algorithm widely used in the area of image classification. SVM is capable of handling multiclass classification problems. SVM classifier creates a hyperplane that separates the possible outputs:

2 $${W}^{T}X-b=0,$$

where *W^T* is the transpose of the weight vector, which is a vector of coefficients that determines the orientation of the decision boundary in the feature space. The weight vector *'W'* is determined by the optimization algorithm used to train the SVM. *'X'* is a feature vector, which represents a data point in the feature space. *'b'* is the bias term, which determines the position of the decision boundary in the feature space [17, 18].

- C) Random forest

Random forest is used to predict the outcome based on the target value set from the dataset. It is based on a supervised machine learning algorithm:

3 $$f=\frac{1}{B}\sum\nolimits_{{b - 1 \to B}}fb\left({x}^{^{\prime}}\right)$$

where *'b'* is the number of decision trees in the forest. *fb(x')* is the prediction of the bth decision tree on the input feature vector *x'* and *1/B* is the scaling factor that averages the prediction of all the decision trees [18].

### Deep learning models used

- A) Artificial neural network

A neural network is a set of algorithms that are used to extract information and underlying relationships between the data in the MRI dataset. It works in a similar way a human brain works. There are layers of interconnected neurons in the neural network; these neurons are known as perceptrons. These perceptrons are mathematical functions that classify information from the MRI image dataset according to the requirements and specific architecture.

A neural network generally consists of two layers only; this is not a suitable condition for the computation of large networks such as MRI images. So for deep learning, we introduce more layers to the traditional neural network. It ranges from 10 to 100 layers based on the computation network. Each neuron in the layers stores some information and passes that information to the forward neurons. As this data flows from the network, the hidden information is extracted from the MRI images. Lower layer neurons generally collect the raw data.

4 $$\sum\nolimits_{{i - 0 \to n}}Wi*Xi+b.$$

The above equation represents the output of a single neuron in ANN. Where n indicates the inputs to the neuron, *Wi* is weight and *Xi* is the value associated with the ith input, and *‘b’* is the bias term. It computes weighted sum of the inputs, adds a bias term, and then applies the activation function to produce the output of the neuron [19, 20].

- B) Convolutional neural networks (CNN)

In a convolutional network, 2 images represented in the matrix form are multiplied to create a new matrix and from that output, features are extracted. There are two processes that a CNN performs first if feature extraction and second classification. The major advantage of using CNN is the ability to learn and generalize the features from a large data set.

5 $$fx,y\left(S\right)=\underset{a,b=0}{\mathrm{max}}S2x+a,2y+b.$$

The above equation represents a type of pooling operation. In CNN, the input image is convolved with a set of learnable filters to produce a set of feature maps. After convolution operation, a pooling layer is applied to reduce the spatial dimension of the feature maps and extract the most important features. In the equation *'a'* and *'b'* represents the horizontal and vertical offsets of the subregion from the top-left corner at position (2x, 2y). The value of *'a'* and *'b'* can either be '0' or '1' which determines the position of the bottom-right corner of the subregion relative to (2x, 2y).

Research articles included in this review paper are using various CNN models such as, ResNet-101, VGG, VoxCNN, DenseNet a brief description of following CNN models are as follows:

- **ResNet-10**1: ResNet-101 is one member of a family of ResNet models, including ResNet-50, ResNet-152, and others, with the number indicating the total number of layers in the network. The input to ResNet-101 is an RGB image with a resolution of 224 × 224 pixels, and the first layer is a convolutional layer with 7 × 7 filters, followed by a batch normalization layer and a ReLU activation function. ResNet-101 then has a series of residual blocks, each consisting of two or three convolutional layers with batch normalization and ReLU activation functions. The architecture has a total of 101 layers. The final layers of ResNet-101 include a global average pooling layer that takes the average of each feature map, a fully connected layer, and a softmax activation function that produces the output classification probabilities [18–20].
- **VGG**: VGG is a 2D convolutional neural network (CNN) architecture that is primarily used for image classification. The input to the VGG model is an RGB image with a resolution of 224 × 224 pixels. The network consists of a series of convolutional layers, each of which has small 3 × 3 filters. The number of filters in each layer increases with the depth of the network. Following each convolutional layer, VGG applies the rectified linear unit (ReLU) activation function to introduce nonlinearity into the model. After each set of convolutional layers, VGG uses a max pooling layer with a pool size of 2 × 2 to reduce the spatial size of the feature maps and increase translation invariance. Finally, the output layer of VGG produces the output classification probabilities [18–20].
- **VoxCNN**: VoxCNN is a powerful 3D convolutional neural network architecture specifically designed to process 3D volumetric data such as medical images and videos. The network includes convolutional layers, activation functions, max pooling layers, dropout layers, and fully connected layers. Each convolutional layer has a small filter size of 3 × 3x3, and the number of filters increases with the depth of the network. Max pooling layers with a pool size of 2 × 2x2 are applied after each convolutional layer to capture the spatial and temporal features of the data. Dropout layers are also used to prevent overfitting during training by randomly dropping out some neurons. The output layer uses the softmax activation function to produce the final classification probabilities. Overall, VoxCNN is a highly effective architecture for various applications, including medical image analysis and video processing.
- **DensNet:** The main novelty of the DenseNet architecture is the use of dense connections between layers, which enables efficient information flow through the network. The first layer of the network is a standard convolutional layer with a 7 × 7 filter, followed by batch normalization and ReLU activation function. The network consists of several dense blocks, each containing multiple dense layers where each layer passes the input through batch normalization, ReLU activation function, and a 3 × 3 convolutional layer with a fixed number of filters. In a dense block, the output of each dense layer is concatenated with the outputs of all previous layers in the same block, allowing each layer to access the previous layer's outputs for efficient information flow. Max pooling layers are used after each set of layers to reduce the spatial size of the feature maps and introduce translation invariance, while dropout layers prevent overfitting during training. DenseNet also includes transition layers between each pair of dense blocks, consisting of a batch normalization layer, a 1 × 1 convolutional layer to reduce the number of feature maps, and a 2 × 2 average pooling layer to down sample the spatial size of the feature maps. Finally, the network ends with a global average pooling layer, a fully connected layer, and a softmax activation function for producing output classification probabilities [20, 21].

C) Recurrent neural network (RNN)

RNN is used in the effective prognosis of Alzheimer’s disease. RNN is used to predict the future health of the patient suffering from AD; also it predicts whether an MCI patient will convert into AD or not based on the clinical data of a patient at different time stamps fed into it. Nguyen et al. in august 2020 proposed a minimal-RNN and fed it with patent data taken at three different time stamps and predict the state for the next three time stamps. This data was collected from ADNI [22].

The formula for current state:

6 $${h}_{t}=f\left({h}_{t-1},{x}_{t}\right).$$

Based on the previous state *h_(t-1)* and current input *x_t*, a recurrent neural network (RNN) calculates its current state *h_t*. An RNN processes a sequence of inputs *x_1*, *x_2*,…, *x_T* and outputs *y_1, y_2,…, y_T*. The non-linear transformation *'f'* maps inputs and previous state to current state.

The formula for activation function:

7 $${h}_{t}=\mathrm{tanh}\left({W}_{hh}{h}_{t-1}+{W}_{xh},{X}_{t}\right).$$

The activation function in a recurrent neural network (RNN) computes the current state *h_t* based on the previous state *h_(t-1)* and the current input *x_t*. The non-linear hyperbolic tangent activation function (tanh) maps its input to a value between -1 and 1. RNNs use the hyperbolic tangent activation function because of its benefits. The output is always finite because it is bounded. Second, it is differentiable, enabling backpropagation network training. Third, it is symmetric around the origin, reducing the RNN vanishing gradient problem.

The formula for output:

8 $${h}_{t}={W}_{hy}{h}_{t}.$$

In the given equation, h_t is the hidden state vector of the RNN at time step t, W_hy is the weight matrix that maps the hidden state to the output vector y, and y_t is the output of the RNN at time stamp t.

**Fig. 3:**
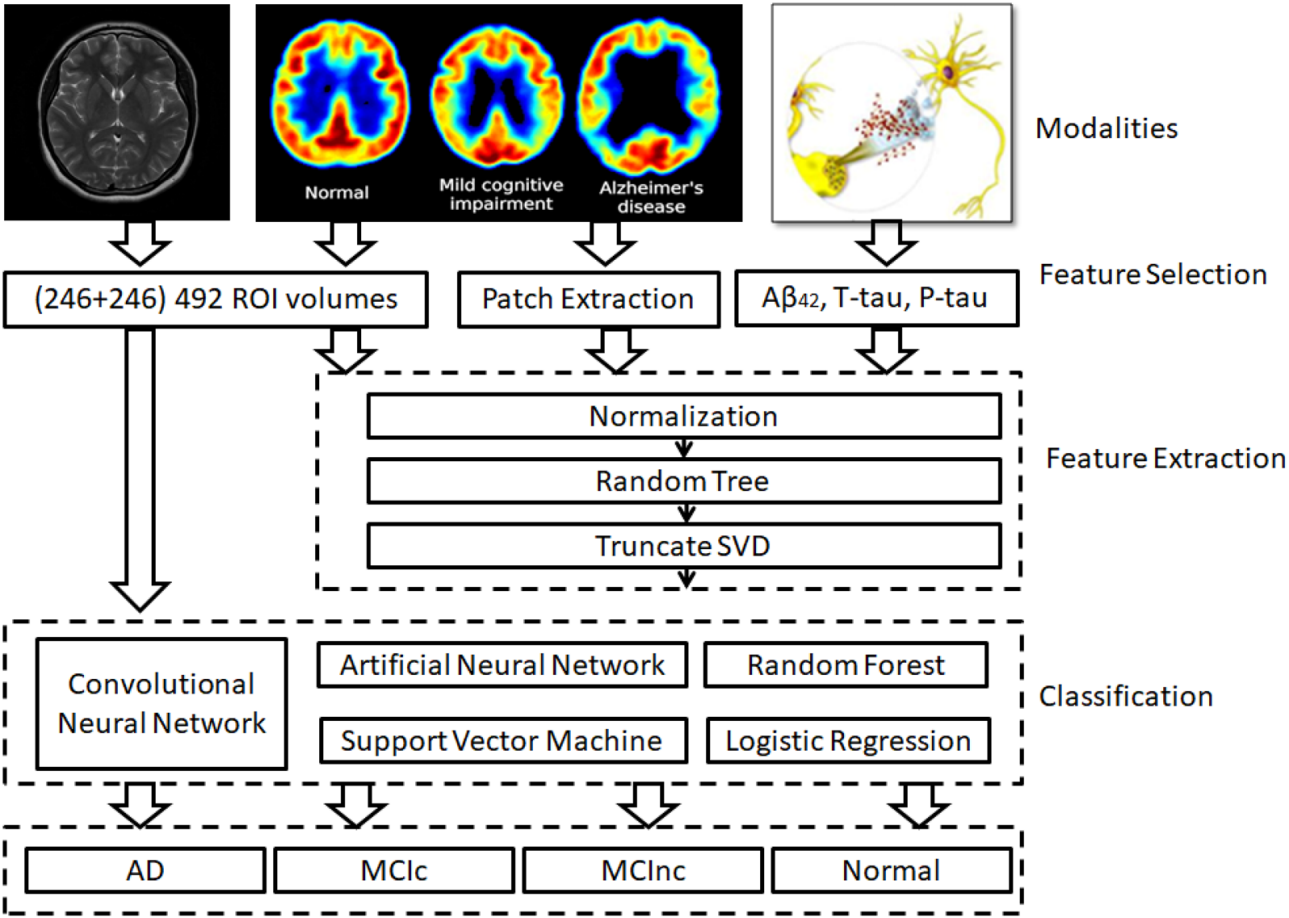
Description of modalities, feature selection, feature extraction, and classification algorithms used to predict AD and NC cases

Figure 3 provides an overview of the modalities, feature selection methods, feature extraction techniques, and classification algorithms employed in the reviewed research articles. The predominant modalities utilized by researchers include fMRI images, PET images, and cerebrospinal fluid analysis. In the feature selection process, physicians rely on brain imaging to identify areas of interest or patches depicting brain tissue deterioration associated with AD. Two biomarkers, namely p-tau and t-tau, play a vital role in the assessment of the presence and progression of Alzheimer's disease (AD). These biomarkers offer valuable insights into the pathological changes that occur in the brain associated with the disease.

Subsequently, features are extracted from the different modalities using methods such as normalization, random tree, and truncated singular value decomposition (SVD). These techniques effectively reduce the dimensionality of the data while retaining critical information. The extracted features are then employed to train machine learning models for the classification of AD and normal cases. The classification algorithms found in the reviewed articles include convolutional neural networks, artificial neural networks, support vector machines, logistic regression, and random forest.

### Performance evaluation measures

**Fig. 4:**
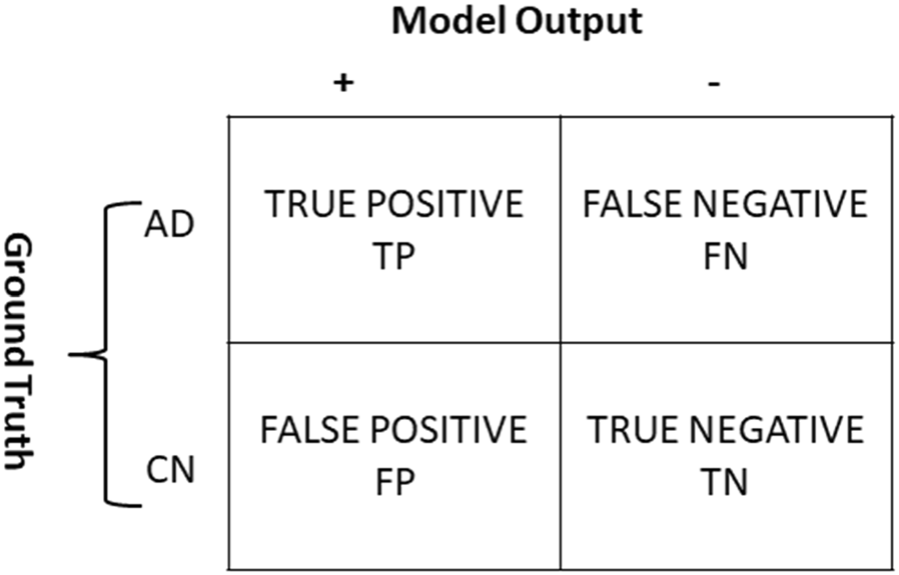
Confusion matrix for the prediction of Alzheimer’s disease

To evaluate the performance of the classification model, a confusion matrix is used. Figure 4 shows the confusion matrix. A confusion matrix is an NxN matrix, where N represents the number of classes in the dataset. In our case, as we are doing binary classification the value of N is 2, positive or negative.

Different parameters such as accuracy, specificity, and sensitivity can be calculated for a model using the confusion matrix:

- Accuracy

Classification accuracy shows the correctness of the prediction by a model in the case of AD diagnosis and how many MRIs are correctly labeled out of the total MRI provided as an input to the model [21]:

9 $$Accuracy=\frac{TP+TN}{TP+FP+TN+FN}.$$

- Sensitivity

Sensitivity is the ratio of correctly positive labeled AD records to all who are suffering from AD in reality [21]:

10 $$Sensitivity=\frac{TP}{TP+FN}.$$

- Specificity

Specificity is the ratio of correctly labeled negative AD records by the model to all who are cognitively normal in reality [21]:

11 $$Specificity=\frac{TN}{FP+TN}.$$

- Precision

Precision is the ratio of the correctly positive labeled AD records by the model to the entire positive labeled by the model [15]:

12 $$Precision=\frac{TP}{TP+FP}*100.$$

- Area under the curve (AUC)-receiver operating characteristics (ROC) curve

AUC-ROC curve ROC is the probability curve and AUC represents the degree that which good a model separates the cases. In our case, it tells how much the model can distinguish between AD and NC. If the AUC is higher the capability of the model to identify the AD and NC cases will increase [15].

## Experimental

Table 2 represents the various research articles included in the study. It also explains the modality and number of subjects included in the research they conducted. Table 3 explains different methods used by the researchers for the diagnosis of AD and various parameters such as accuracy, sensitivity, specificity, and precision achieved with the different models of machine learning.

**Table 2:** Represents the various research articles included in the study. It also explains the modality and number of subjects included in the research they conducted

References	Database	Modality	No. of records				Total
			AD	cMCI/PMCI	ncMCI/SMCI	NC	
Zhang D et al. [3]	ADNI	MRI (T1 weighted), FDG-PET	**38**	**–**	**–**	**50**	**88**
Suk H et al. [4]	ADNI	fMRI TR2	**12**	**–**	**–**	**25**	**37**
Lu D et al. [6]	ADNI	MRI 1.5 T, FDG-PET	**238**	**217**	**409**	**360**	**1224**
Tong T et al. [7]	ADNI	MRI 1.5 T	**198**	**167**	**238**	**231**	**834**
Lee G et al. [9]	ADNI	MRI 1.5 T, CFS, Cog. Score	**338**	**307**	**558**	**415**	**1618**
Xiao Z et al. [10]	ADNI	MRI 1.5 T	**54**	**–**	**542**	**58**	**654**
Ortiz A et al. [12]	ADNI	MRI 1.5 T,FDG-PET	**70**	**26**	**111**	**68**	**275**
Subramoniam M et al.(a) [14]	Kaggle	MRI 1.5 T	**960**	**–**	**2240**	**3200**	**6400**
Subramoniam M et al.(b) [14]	Kaggle	MRI 1.5 T	**960**	**–**	**2240**	**3200**	**6400**
Yue L et al.(a) [23]	ADNI-1	MRI 1.5 T	**335**	**–**	**542**	**785**	**1662**
Yue L et al.(b) [23]	ADNI-2	MRI 3 T	**366**	**1304**	**1583**	**1106**	**4359**
Goryawala M et al. [24]	ADNI	MRI 1.5 T	**55**	**91**	**114**	**125**	**385**
Aderghal [25]	ADNI	MRI 1.5 T	**188**	**–**	**399**	**228**	**815**
Korolev (a) [26]	ADNI	MRI 1.5 T	**50**	**43**	**77**	**61**	**231**
Korolev (b) [26]	ADNI	MRI 1.5 T	**50**	**43**	**77**	**61**	**231**
Cheng and Liu [27]	ADNI	FDG-PET	**93**	**–**	**146**	**100**	**339**
Choi H et al. [28]	ADNI	FDG and AV-45 PET	**139**	**79**	**92**	**182**	**492**
Cheng and Liu [29]	ADNI	MRI 1.5 T, FDG-PET	**93**	**–**	**–**	**100**	**193**
Kumar N et al. [30]	OASIS	MRI 1.5 T	**146**	**–**	**37**	**190**	**373**
Ji h et al. [31]	ADNI	MRI 1.5 T	**179**	**–**	**–**	**182**	**361**
Battineni G et al. [32]	OASIS	MRI 1.5 T	**146**	**–**	**37**	**190**	**373**
Kumari R et al. [33]	OASIS-3	MRI 1.5 T	**489**	**–**	**–**	**609**	**1098**
Alickovic E et al. [34]	ADNI	MRI 1.5 T	**72**	**–**	**–**	**195**	**267**
Madiwalar S et al. [35]	OASIS	MRI 1.5 T	**64**	**–**	**–**	**72**	**136**
Pan D et al. [36]	ADNI	MRI 1.5 T	**237**	**–**	**–**	**262**	**499**
Alroobaea R et al. (a) [37]	ADNI	MRI 1.5 T	**1731**	**–**	**–**	**2665**	**4396**
Alroobaea R et al. (b) [37]	OASIS	MRI 1.5 T	**146**	**–**	**37**	**190**	**373**
Savita et al. [38]	OASIS	MRI 1.5 T	**100**	**–**	**–**	**135**	**235**
Li and Yang [39]	ADNI	MRI 1.5 T	**260**	**–**	**–**	**300**	**560**
Mehmood A et al. [40]	ADNI	MRI 1.5 T	**75**	**–**	**–**	**85**	**160**

**Table 3:** Explains different methods used by the researchers for the diagnosis of AD and various parameters such as accuracy, sensitivity, specificity, and precision achieved with the different models of machine learning.

References	Method	AD:NC Acc	SEN recall	SPE	Precision	AUC
Zhang D et al. [3]	Multi-kernel SVM	**78.40**	**79.00**	**78.00**	**–**	**76.80**
Suk H et al. [4]	Group sparse representation + SVM	**89.19**	**91.00**	**88.00**	**–**	**95.60**
Lu D et al. [6]	DNN	**82.93**	**79.69**	**83.84**	**–**	**–**
Tong T et al. [7]	Multiple instance learning + SVM	**89.00**	**–**	**–**	**–**	**–**
Lee G et al. [9]	Multi- model deep learning + RNN	**81.00**	**84.00**	**80.00**	**–**	**86.00**
Xiao Z et al. [10]	SVM-REF with covariance	**85.71**	**79.63**	**91.38**	**–**	**–**
Ortiz A et al. [12]	Deep belief network (SVM)	**90.00**	**86.00**	**94.00**	**–**	**95.00**
Subramoniam M et al.(a) [14]	Vanilla DNN	**95.31**	**–**	**–**	**–**	**–**
Subramoniam M et al.(b) [14]	CNN	**95.32**	**–**	**–**	**–**	**–**
Yue L et al.(a) [23]	CNN	**99.40**	**100**	**–**	**98.80**	**97.20**
Yue L et al.(b) [23]	CNN	**98.60**	**97.20**	**–**	**100**	**98.60**
Goryawala M et al. [24]	Linear regression model + LDA	**93.90**	**96.30**	**89.50**	**93.80**	**–**
Aderghal [25]	2D CNN	**91.41**	**93.75**	**89.60**	**–**	**–**
Korolev (a) [26]	3D CNN	**79.00**	**–**	**–**	**–**	**88.00**
Korolev (b) [26]	ResNet	**80.00**	**–**	**–**	**–**	**87.00**
Cheng and Liu [27]	RNN	**91.20**	**91.40**	**83.84**	**95.30**	**–**
Choi H et al. [28]	3D CNN	**96.00**	**–**	**–**	**–**	**91.00**
Cheng and Liu [29]	3D CNN	**89.60**	**87.10**	**92.00**	**–**	**94.45**
Kumar N et al. [30]	Linear discriminant analysis	**98.92**	**–**	**–**	**–**	**–**
Ji h et al. [31]	CNN	**98.59**	**97.22**	**100**	**–**	**–**
Battineni G et al. [32]	Linear regression model	**98.30**	**97.40**	**–**	**98.60**	**99.70**
Kumari R et al. [33]	CNN	**90.25**	**85.53**	**–**	**–**	**–**
Alickovic E et al. [34]	Random forest	**85.77**	**54.17**	**97.44**	**–**	**–**
Madiwalar S et al. [35]	Extra tree	**93.14**	**85.00**	**–**	**85.00**	**–**
Pan D et al. [36]	CNN	**84.00**	**–**	**–**	**–**	**92.00**
Alroobaea R et al. (a) [37]	Random forest	**98.89**	**99.19**	**–**	**98.89**	**–**
Alroobaea R et al. (b) [37]	Logistic regression	**84.33**	**84.14**	**–**	**84.54**	**–**
Savita et al. [38]	SVM	**85.80**	**–**	**–**	**87.83**	**–**
Li and Yang [39]	SVM	**90.00**	**93.90**	**85.10**	**–**	**97.00**
Mehmood A et al. [40]	CNN	**98.73**	**98.19**	**99.09**	**–**	**–**

Based on the study it is evident that Deep Learning techniques for feature extraction and the traditional machine learning approach of classification using a support vector machine (SVM) classifier are highly efficient in the diagnosis and prediction of Alzheimer’s disease. It has also been observed that multimodality-based diagnosis and prognosis perform better than single modality-based techniques. These can include clinical tests and cognitive tests [41, 42].

**Fig. 5:**
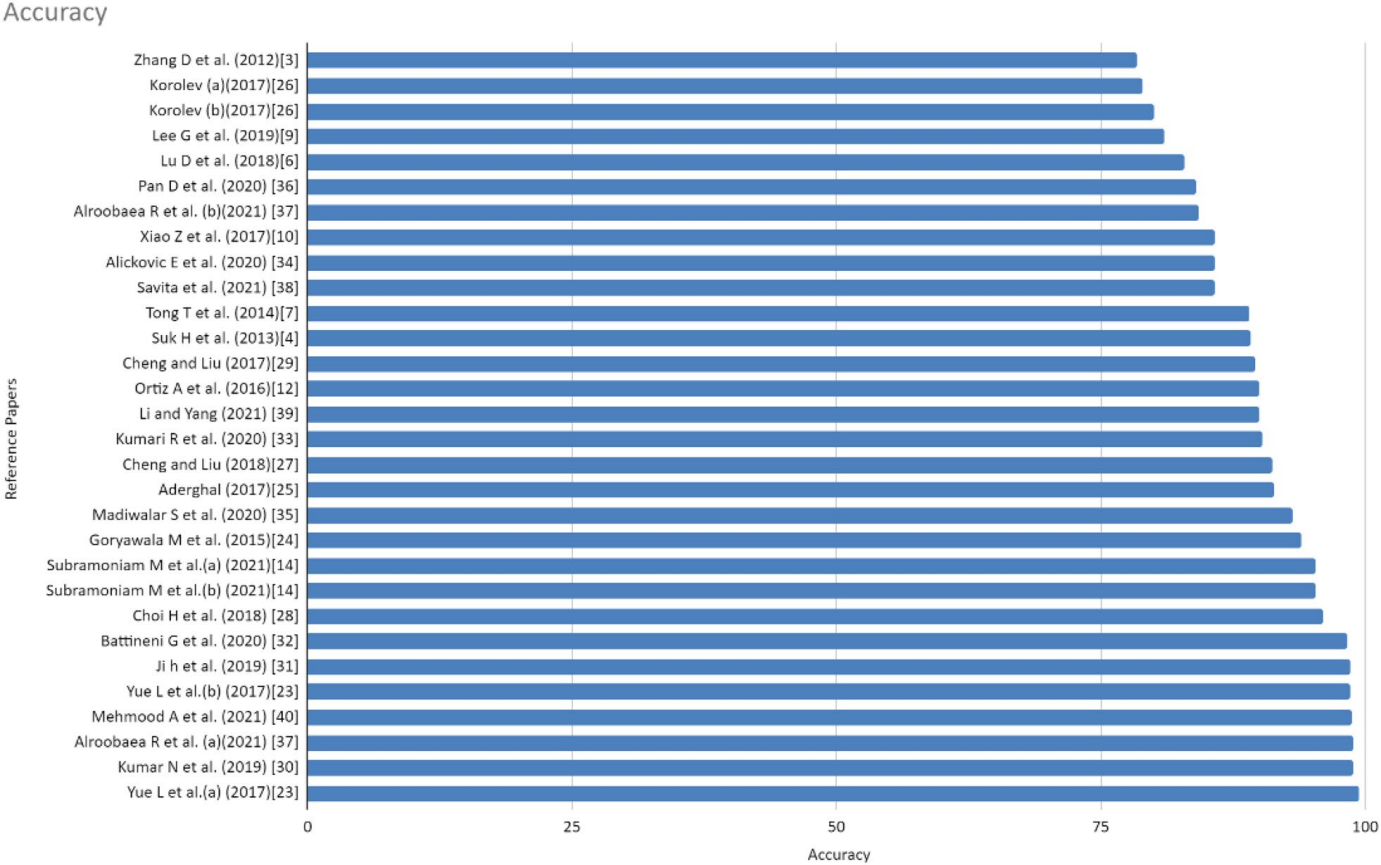
Comparison chart of accuracy derived from different articles

A voxel-based feature extraction method for the early diagnosis of AD plays a pivotal role. In this approach, the brain image is divided into 90 regions of interest (ROI) out of which only informative voxels are selected and stored into a vector for the study corresponding to the baseline. These voxels were provided as input to CNN and variants of CNN such as ensemble system of deep convolutional neural networks and Siamese convolutional neural network [43, 44] for further deep learning. The robustness of the system is then tested against the subset from ADNI. Figure 5 shows that it achieved the accuracy of 97.2% in the case of cognitive normal to AD and 99.4% in the case of MCI-NC to MCI-C. The total subjects included in the study are 1662 which includes 785 cognitively normal, 542 MCI, and 335 AD cases.

The development of a framework to assess the progression of AD using MRI volumes and neuropsychological scores is also a significant approach to the diagnosis and prognosis of Alzheimer’s disease. In this framework using the features selection method subjects were divided into two groups, early MCI (EMCI) and late MCI (LMCI), effectively with an accuracy rate of 73.6%. This results in a significant amount of improvement and the accuracy achieved with this model is 93.9% in the case of NC/AD classification which can be observed in Fig. 5.

**Fig. 6:**
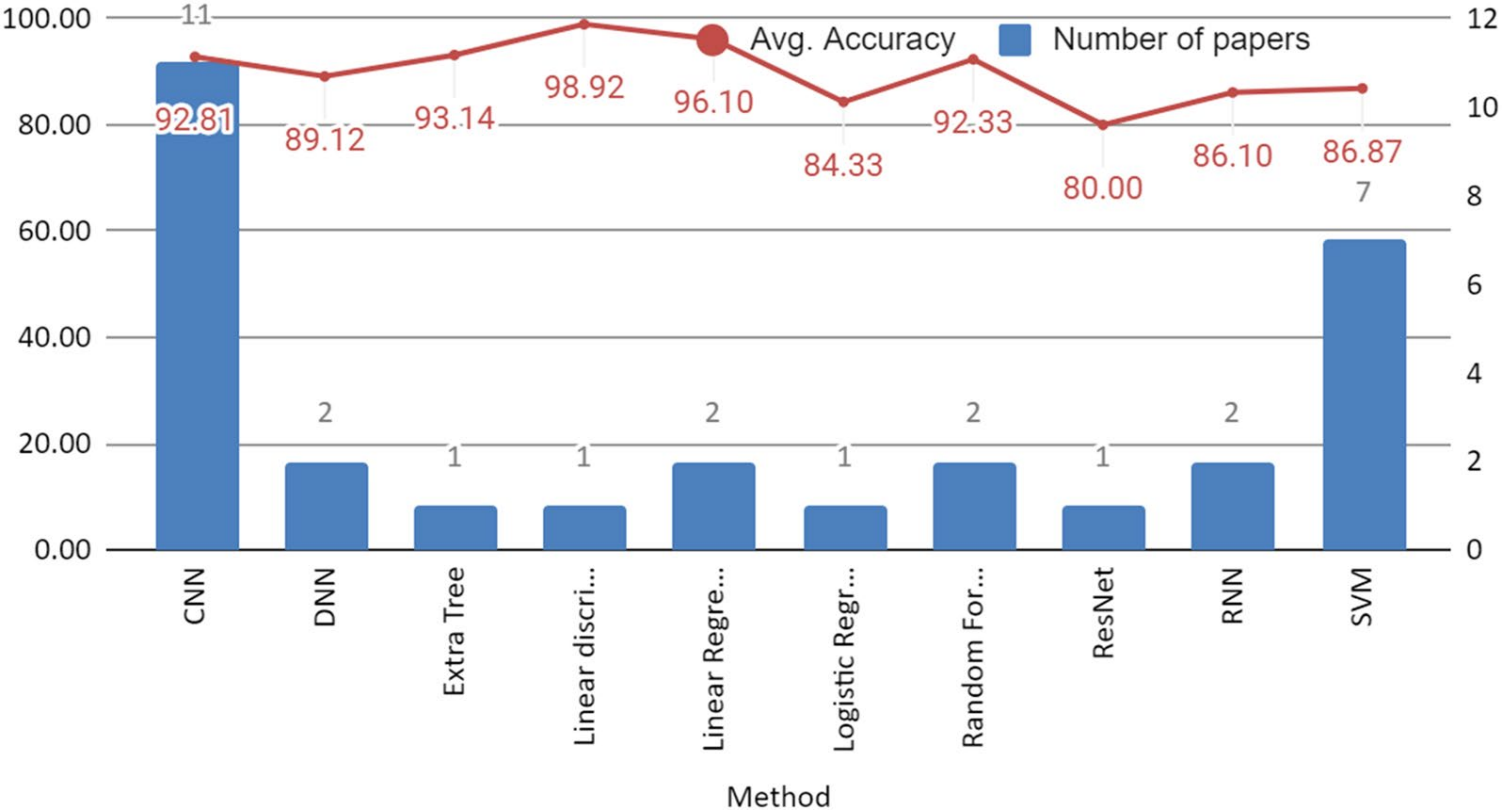
Frequency of methods used in different articles taken in the study, and average accuracy using different classifiers

Figure 6 illustrates two primary pieces of information. Firstly, it presents the frequency of usage of machine learning and deep learning algorithms by researchers. Secondly, it showcases the highest accuracy achieved by each specific algorithm. It is evident that there is a prevalent trend of employing deep learning methods, which can be attributed to their notable accuracy, particularly in handling complex three-dimensional data. Deep learning models have demonstrated superior performance in the domain of Alzheimer's disease research, making them a preferred choice for many researchers.

**Fig. 7:**
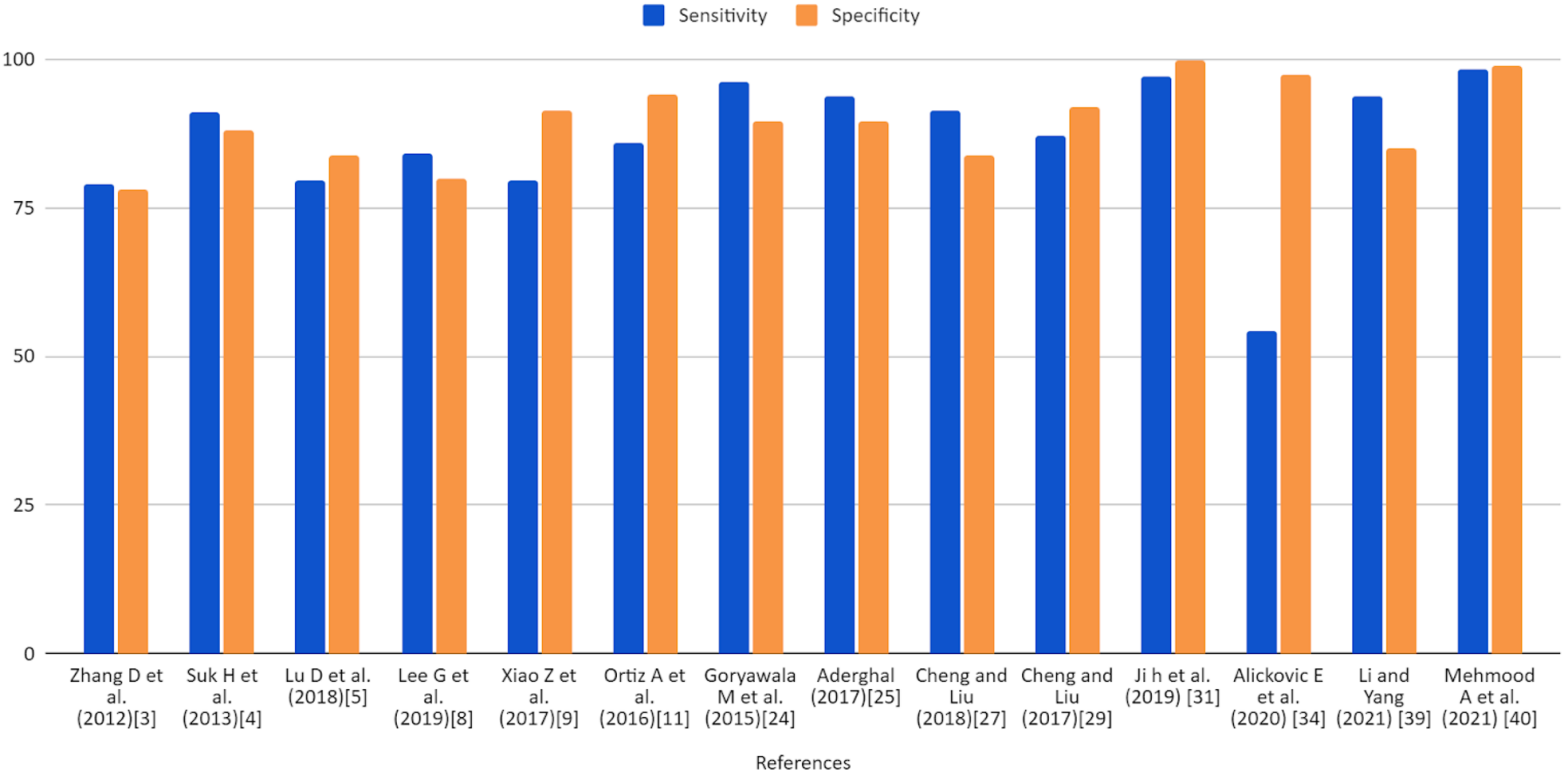
Comparison chart of performance in terms of sensitivity and specificity

**Fig. 8:**
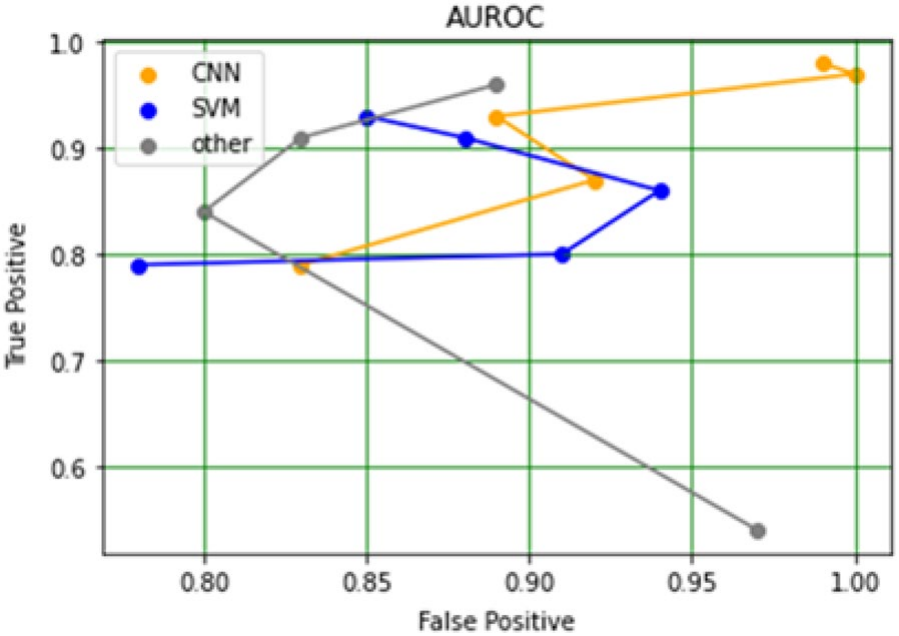
AUROC curve between the true positive rate and false positive rate derived from CNN, SVM, and, other methods

**Fig. 9:**
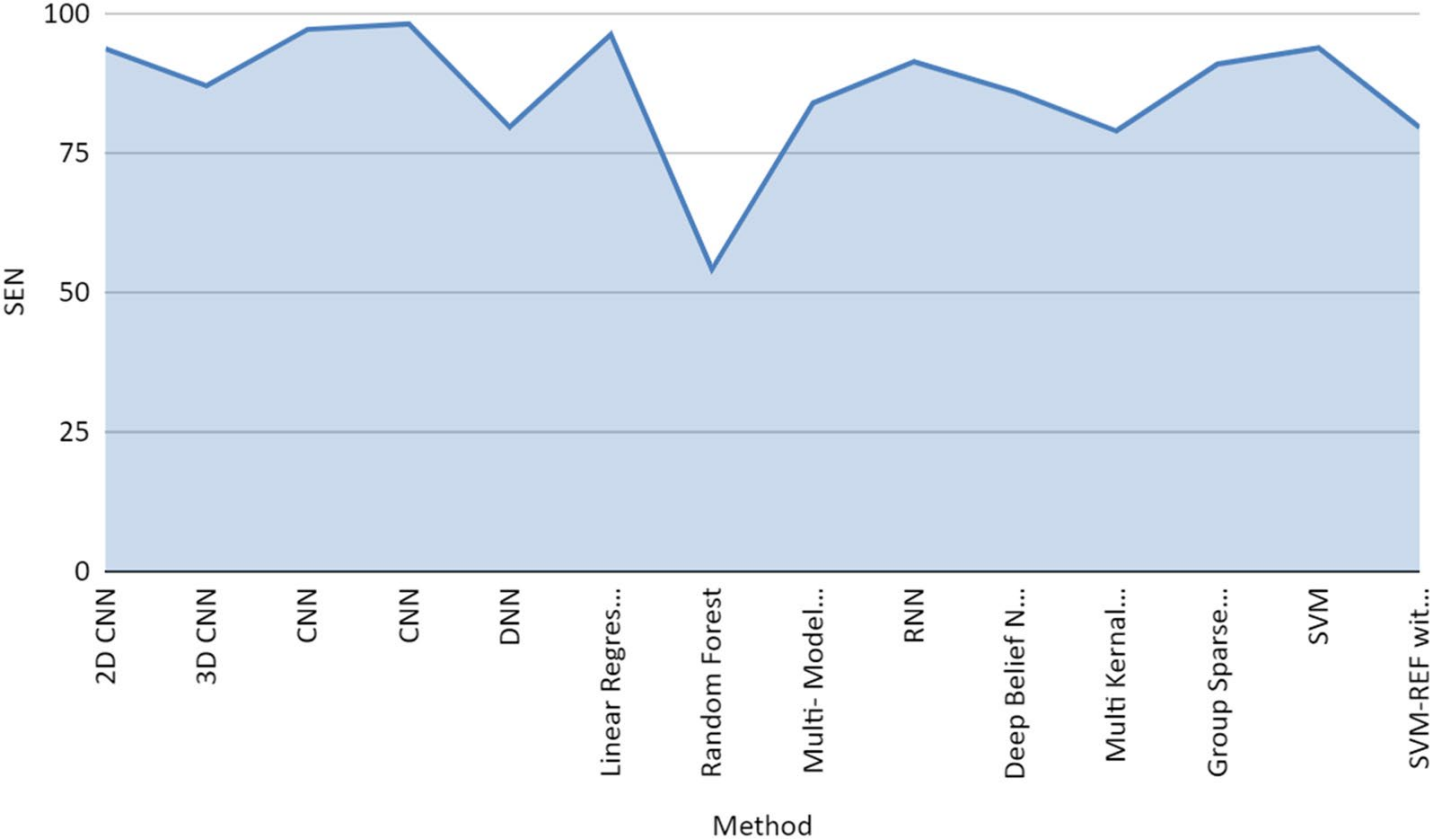
Stacked area chart of the sensitivity drive from different methods used in the study

Figure 7 represents the performance achieved by different research articles in terms of sensitivity (true positive rate) and specificity (false positive rate). The true positive rate indicates that the model predicts the positive cases correctly. And the False positive rate indicates that the model predicts negative to the positive class. Figure 8 is the AUROC curve between true positive rate (TPR) and false positive rates (FPR) achieved by CNN, SVM, and other methods used in the research articles included in the study. Figures 8 and 9 represent the stacked area chart of the TPR and FPR.

**Fig. 10:**
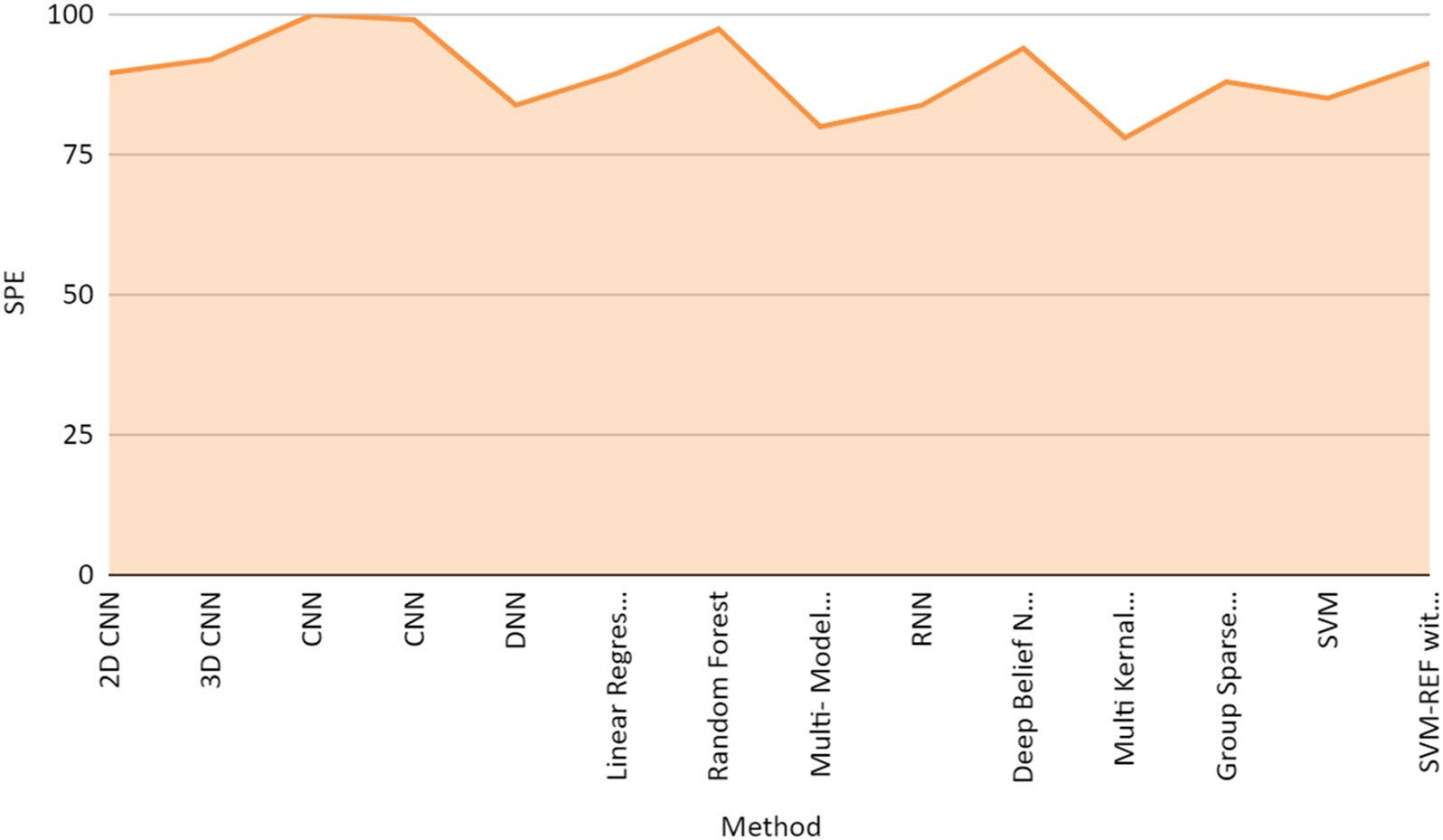
Stacked area chart of the specificity drive from different methods used in the study

Figures 9 and 10 are the stacked representation of the sensitivity and specificity derived from different methods used by research articles referred to in this review paper. Application of 3d CNN with PET images achieved an accuracy of 96% for AD/NC classification and accuracy of 84.2% in the case of MCI-C/ MCI-NC which is much better in comparison to results achieved by SVM classifiers. CNN for the classification achieved an accuracy of 91.41% while RNN on PET images achieved an accuracy of 91.2% in NC/ AD classification. If the landmark detection method with CNN on MRI images was applied then it achieved an accuracy of 91.09%.

## Limitations

It is crucial to acknowledge the limitations of this study. While there are various modalities available for the diagnosis of Alzheimer's disease, including medical history, blood tests, spinal taps, and genetic testing, this study only examines research articles that utilize two specific modalities: MRI/PET scans and cognitive assessments obtained from clinical data sets.

AD patients often exhibit alterations in brain electrical activity, which can be reflected in magnetic field measurements. Magnetometer and gradiometer data, researchers have the opportunity to capture and analyze magnetic field variations, allowing for the identification of potential patterns or signatures that differentiate AD patients from healthy individuals [45].

Additionally, it is important to note that the literature reviewed in this study heavily relies on data from the Alzheimer's disease Neuroimaging Initiative (ADNI) database, resulting in a substantial overlap in sample populations. This dependency exists due to the high expense of fMRI data acquisition and limited access to other databases.

## Conclusion

Early and effective diagnosis of Alzheimer’s disease is very critical in the treatment of the patient. With the early detection of AD, it is possible to minimize the effects of AD on neuron degeneration. It is evident that machine learning and deep learning techniques contribute significantly toward the early detection of Alzheimer’s disease. In traditional ML techniques using the support vector machine (SVM) classifiers, an accuracy of 85.71% can be achieved in the classification of CN/AD. In deep learning using CNN classifier accuracy of 98.6% and with RNN accuracy of 91.2% can be achieved. Our study suggests that decisions made on the predictions based on the multimodalities are more reliable and accurate in comparison of the single modality. This article will be of great assistance in gaining an understanding of the various research approaches that have been utilized in recent studies on Alzheimer's disease.

Recent trends show that use of the deep learning techniques in the analysis of medical images has increased for faster analysis and better accuracy than a human practitioner. This study will facilitate better accuracy in the future by identifying various combinations of existing models. Ensemble learning techniques can enhance the predictive accuracy of better-performing models by combining them. This involves training multiple models on the same dataset and aggregating their predictions to generate a final output. Ensemble learning can provide benefits, such as improving accuracy, mitigating overfitting, and increasing robustness. By leveraging the strengths of multiple models, ensemble learning can help produce more accurate and reliable results.

Future research can expand on the current literature survey and the limitations mentioned in this study. It is recommended that future research must explore additional modalities not covered in the current study. Additionally, leveraging ensemble learning techniques to combine results from multiple models may be beneficial in investigating the early stages of Alzheimer's disease [46]. Further Explainable AI (XAI) can be used for the explanation on the reliability and stability of the model used. XAI techniques aims to provide transparent and interpretable insights into the decision-making processes of machine learning models, particularly in complex domains such as AD diagnosis and classification [47]. To overcome the challenge of limited sample size, multiple neuroimaging databases or transfer learning could be utilized. Overall, future studies should prioritize achieving robust and accurate outcomes.

## Data Availability

Open-source databases accelerate research work in the field of diagnosis and prognosis of Alzheimer’s diseases by providing neuroimages. Some of the most common and reliable databases are Alzheimer’s disease neuroimaging initiative (ADNI) and OASIS.
